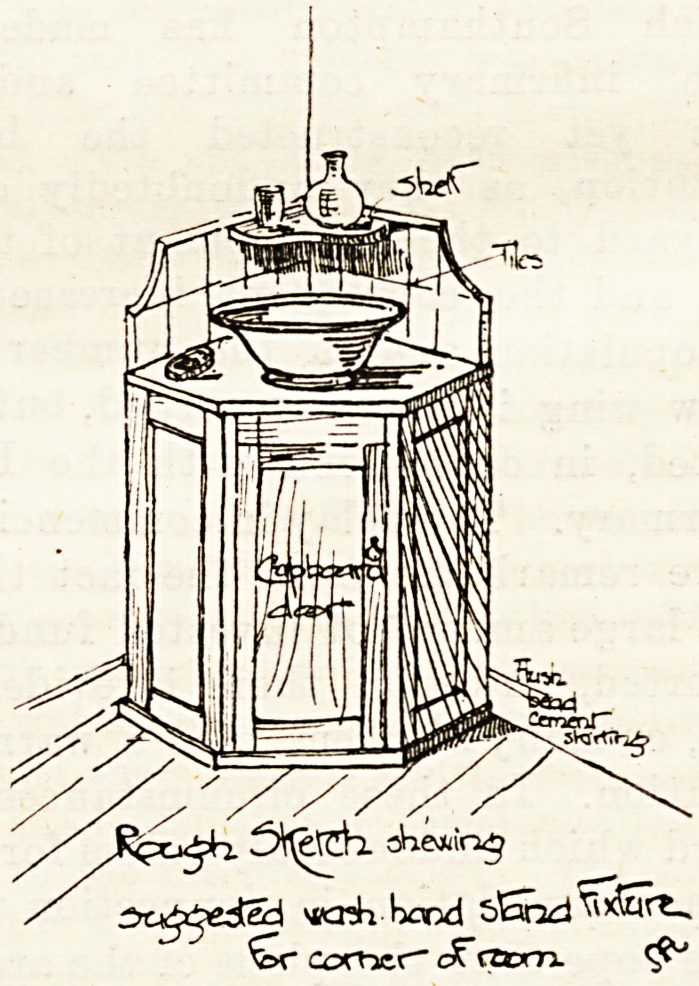# Furniture and Fittings for Nurses' Homes

**Published:** 1895-09-28

**Authors:** 


### PRACTICAL DEPARTMENTS.

### I.—FURNITURE AND FITTINGS FOR NURSES' HOMES.

The " Nurses' Home," properly so-called, now to be found attached, in most cases a separate building, to every hospital or infirmary of any size or importance, has been a natural consequence of the enormous advances made in the training of nurses during the last half of the present century. The century now so near its end will certainly be remembered in future for the creation of the profession of nursing, and to meet the wants of its members very different arrangements to those which sufficed twenty or even ten years ago have become essential. Then when a hospital was in course of erection bed-rooms for nurses were generally provided on the top storey. Dining and sitting rooms did not exist; the nurses and probationers took their meals when and how they could, and, as for recreation—well, hospital committees certainly did not feel themselves called upon to provide the comfortable quarters, fitted with easy chairs and sofas, for the nurses of that period which fall to the lot of their sisters of to-day.

Now, it is not too much to say that when a new hospital is about to be erected, the adequate planning and arranging of the nurses' home is one of its most important and well-considered features, and hospital authorities vie with each other in endeavouring to secure the greatest comfort for their nursing staff. It may not be uninteresting, therefore, to compare and see what are some of the plans followed in certain of the modern hospitals towards the attainment of this end. But before giving details of the arrangement of the nurses' quarters in these places, we would say there are certain points to be considered in regard to fittings.

To begin with, wherever possible, as in the case of the building of a new home, separate bed-rooms, rather than cubicles or double-bedded rooms, are undoubtedly very necessary. The increase of comfort is immense, as anyone who has had experience of the one and the other can testify. For nurses, above all people, whose nerves must be frequently tried to the utmost, the quiet of the solitary possession of even the tiniest apartment is of the greatest value. For weary women, whose only desire in bed is sleep, to be at the possible mercy of more wakeful companions in neighbouring cubicles is really a hardship. It is, in the next place, very desirable that the actual furniture shall be as much condensed, so to speak, as practicable, to economise floor space to the greatest degree. Very good designs for dressing-tables and wash-stands of the combination type are now to be found, and excellent contrivances have been thought and carried out in some of the more recently planned homes.

There is no doubt that it is an excellent thing in designing plans for a nurses' home to arrange for certain portions of the fittings as fixtures. We have advocated this idea before, and in The Hospital for July 22nd, 1893 (p. 271), will be found a sketch of a proposed plan for fitting up one entire side of each small room with cupboards and drawers from floor to ceiling, the top ones being intended for such articles as may not be in constant use. By this arrangement dust collecting corners and spaces are avoided to a large extent; it would probably prove cheaper in the long run than the purchase of separate wardrobe, chest of drawers, &c., and would be infinitely more convenient to the occupier. If one side of a room were thus disposed of, another corner might be fitted with washstand, leaving plenty of room for bed, chair, and small table. The hanging cupboard door ought to be filled with glass, for a mirror long enough to serve as a reminder that tidy skirts, well off the ground, form an important item in a nurse's attire, is very desirable. We do not know if such a plan as the above has ever been attempted in a nursing home, but it seems to us to be eminently practical, and one which we should be glad to see carried out by way of experiment.

Corners are seldom made the most of, so far as our experience goes. There can be few instances where it would not be cheaper and better to have the dress cupboard fixed across a corner or in a recess, than to fill more space less commodiously with—often—a not too well-made wardrobe with a drawer that declines to open, a door that refuses to shut. In the same way it is a very convenient plan to have the stand for wash-hand basin, &c., a corner fixture, something after the fashion of the accompanying drawing, with cupboard below for boots and shoes and other oddments.

At the Poplar Hospital, where Mr. Sydney Holland has carried through many an ingenious device of his "own invention," an idea of this kind has been put into execution in the matron's rooms. These really are ideal rooms, their decoration is so exceedingly simple and effective withal. To begin with, the sitting-room is panelled to a height of about six feet, the woodwork painted in artistic shades—green—if we remember rightly. Then between the two windows is a delightful three-sided bookcase, forming part of the panelling, as it were. Shelves are carried along in the recess on one side of the fireplace, the whole having a charmingly harmonious and comfortable effect. In the bed-room the same plan has been followed. Chests of drawers, hanging cupboard, and dressing-table are all made practically in one, the cupboard with long glass being in the centre and cutting off a corner, the whole forming a continuous fixture.

(*To be continued.*)

## Figures and Tables

**Figure f1:**